# Curcumin Reverse Methicillin Resistance in *Staphylococcus aureus*

**DOI:** 10.3390/molecules191118283

**Published:** 2014-11-10

**Authors:** Su-Hyun Mun, Sung-Bae Kim, Ryong Kong, Jang-Gi Choi, Youn-Chul Kim, Dong-Won Shin, Ok-Hwa Kang, Dong-Yeul Kwon

**Affiliations:** 1 BK21 Plus Team, Professional Graduate School of Oriental Medicine, Wonkwang University, Iksan, Jeonbuk 570-749, Korea; 2 Department of Oriental Pharmacy, College of Pharmacy, Wonkwang Oriental Medicines Research Institute, Institute of Biotechnology, Wonkwang University, Iksan, Jeonbuk 570-749, Korea; 3 Standardized Material Bank for New Botanical Drugs, College of Pharmacy, Wonkwang University, Iksan, Jeonbuk 570-749, Korea; 4 Department of Oriental Medicine Resources, College of Bio Industry Science, Sunchon National University, Sunchon, Jeonnam 540-742, Korea

**Keywords:** curcumin, methicillin-resistant *Staphylococcus aureus*, antimicrobial, detergents, ATPase inhibitors, peptidoglycan, PBP2a

## Abstract

Curcumin, a natural polyphenolic flavonoid extracted from the rhizome of *Curcuma longa* L., was shown to possess superior potency to resensitize methicillin-resistant *Staphylococcus aureus* (MRSA) to antibiotics. Previous studies have shown the synergistic activity of curcumin with β-lactam and quinolone antibiotics. Further, to understand the anti-MRSA mechanism of curcumin, we investigated the potentiated effect of curcumin by its interaction in diverse conditions. The mechanism of anti-MRSA action of curcumin was analyzed by the viability assay in the presence of detergents, ATPase inhibitors and peptidoglycan (PGN) from *S. aureus*, and the PBP2a protein level was analyzed by western blotting. The morphological changes in the curcumin-treated MRSA strains were investigated by transmission electron microscopy (TEM). We analyzed increased susceptibility to MRSA isolates in the presence of curcumin. The optical densities at 600 nm (OD_600_) of the suspensions treated with the combinations of curcumin with triton X-100 and Tris were reduced to 63% and 59%, respectively, compared to curcumin without treatment. *N,N'*-dicyclohexylcarbodiimide (DCCD) and sodium azide (NaN_3_) were reduced to 94% and 55%, respectively. When peptidoglycan (PGN) from *S. aureus* was combined with curcumin, PGN (0–125 μg/mL) gradually blocked the antibacterial activity of curcumin (125 μg/mL); however, at a concentration of 125 µg/mL PGN, it did not completely block curcumin. Curcumin has a significant effect on the protein level of PBP2a. The TEM images of MRSA showed damage of the cell wall, disruption of the cytoplasmic contents, broken cell membrane and cell lysis after the treatment of curcumin. These data indicate a remarkable antibacterial effect of curcumin, with membrane permeability enhancers and ATPase inhibitors, and curcumin did not directly bind to PGN on the cell wall. Further, the antimicrobial action of curcumin involved in the PBP2a-mediated resistance mechanism was investigated.

## 1. Introduction

The Gram-positive bacterium, methicillin-resistant *Staphylococcus aureus* (MRSA), is resistant to β-lactam antibiotics, because of the lowered β-lactam affinity to penicillin-binding proteins (PBP) and PBP2a (there is one fifth PBP in *S. aureus*, namely, PBP2a), encoded by the *mecA* gene determinant. MRSA has acquired the *mecA* gene, enabling the bacteria to sustain cell-wall synthesis. The emergence of antibiotic resistance genes in *S. aureus* has facilitated the evolution of multidrug-resistant bacteria. The over-prescription of antibiotics is leading to new strains of drug-resistant bacteria [1,2]. MRSA has received significant attention throughout the past few decades, as it is a major cause of nosocomial (hospital acquired) infections. *S. aureus* has generated a resistance to the β-lactam antibiotics, because of the production of chromosomal- or plasmid-mediated β-lactamases [3].

The number of effective antibiotics is decreasing, and therefore, constant effort is required for the identification of antimicrobial materials from natural products and traditional medicines. A number of natural products have been confirmed as new antimicrobial drugs; however, there is still an urgent need to identify novel substances active towards pathogens with a high resistance [4].

We have previously shown that curcumin, a dominant polyphenol compound extracted from the rhizomes of *Curcuma longa* L., potentiates the effects of β-lactam and quinolone antibiotics against MRSA [5]. Curcumin has a number of biological activities, including antibacterial, anti-inflammatory and antitumor effects [6,7,8,9]; however, research on the antimicrobial activity mechanism of curcumin against MRSA has not yet been investigated.

The aim of this study was to investigate the antimicrobial activity of curcumin associated with the reduced viability of bacteria in the combination treatments with membrane permeability agents and ATPase inhibitors. Then, we analyzed the action of curcumin on the peptidoglycan (PGN) derived from the cell wall of *S. aureus* and further determined the curcumin-induced effect on oxacillin resistance in MRSA.

**Figure 1:**
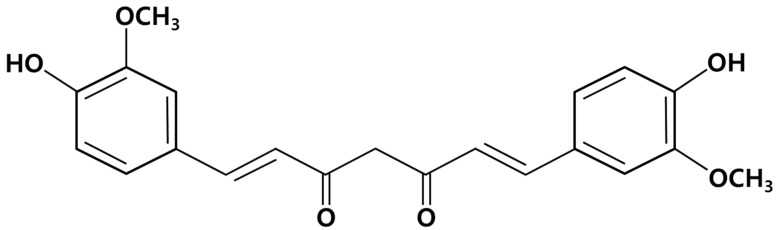
The structure of curcumin.

Curcumin is the main colorant found in the rhizomes of *Curcuma longa* L. and has shown to possess therapeutic effects on injury treatment and skin tumors after topical application (Figure 1) [10]. Curcumin has an antimicrobial effect against many microorganisms, especially with valid activity against *Bacillus subtilis*, *Escherichia coli* and *S. aureus* [11]. Microcapsule curcumin has previously been studied for its antibacterial and antifungal activities against foodborne pathogens and putrefactive bacteria, such as *Escherichia coli*, *Yersinia enterocolitica*, *Saccharomyces cerevisiae*, *Bacillus subtilis*, *Bacillus cereus*, *Aspergillus niger*, *Penicillium notatum* and *S. aureus* [12].

## 2. Results and Discussion

### 2.1. Antibacterial Activity of Curcumin with Detergents or ATPase Inhibitors

**Figure 2:**
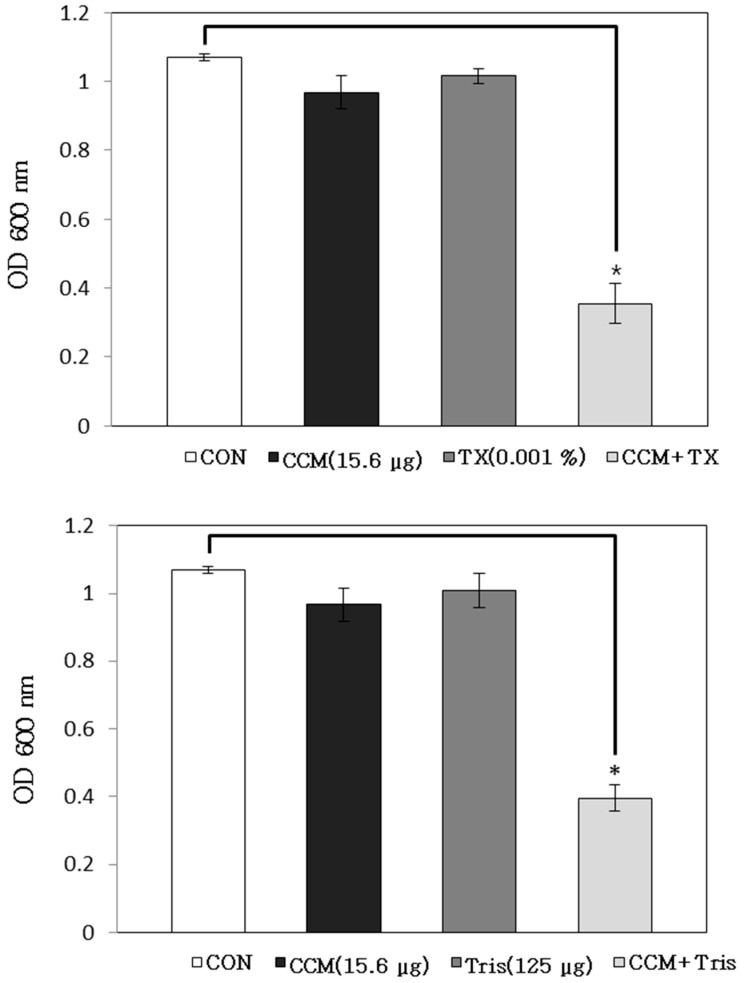
The effects of the membrane-permeabilizing agents Triton X-100 (TX-100) and *tris*-(hydroxymethyl) aminomethane (Tris) on the susceptibility of *Staphylococcus aureus* (ATCC 33591) to curcumin (CCM) treatment. The viability of bacteria was determined via spectrophotometry (optical density at 600 nm, OD_600_) after incubation for 24 h with 15.6 μg/mL CCM with 0.001% TX-100 and 125 µg/mL Tris, respectively. These data represent as the average of three independent experiments. * *p* < 0.05, as compared to CCM alone, were determined. CON is the acronym for the control *S. aureus* strain, which was not treated.

We have previously shown that curcumin increases the susceptibility of *Staphylococcus aureus*. Curcumin in combination with four antibiotics effectively inhibited *S. aureus* growth [5]. In this study, to investigate the effects of enhanced membrane permeability on the activity of curcumin, the antibacterial activity of curcumin under conditions of increased membrane permeability was examined using 0.001% triton X-100 (TX-100) and 125 μg/mL Tris. Both TX-100 and Tris are known to increase the permeability of the outer membrane [13]. Modest reductions in the OD_600_ of curcumin-treated suspensions were noted in combination with TX-100 and Tris when measured 24 h later (Figure 2). Compared to curcumin alone, the OD_600_ of the suspensions read 24 h after the treatment with curcumin and TX-100 and curcumin and Tris reduced to 63% and 59%, respectively. The antimicrobial activity of curcumin increased by increasing the membrane permeability of bacterial isolates, and curcumin was found to exhibit a more potent activity in combination with TX-100 and Tris. Moreover, TX-100 decreased methicillin resistance against *S. aureus* strains, with resistant strains showing the highest increase in antibiotic sensitivity. TX-100 stimulated autolysis and the release of acylated lipoteichoic acid. Moreover, the strains that showed the greatest decline in methicillin resistance released substantially more lipoteichoic acid [14,15].

**Figure 3:**
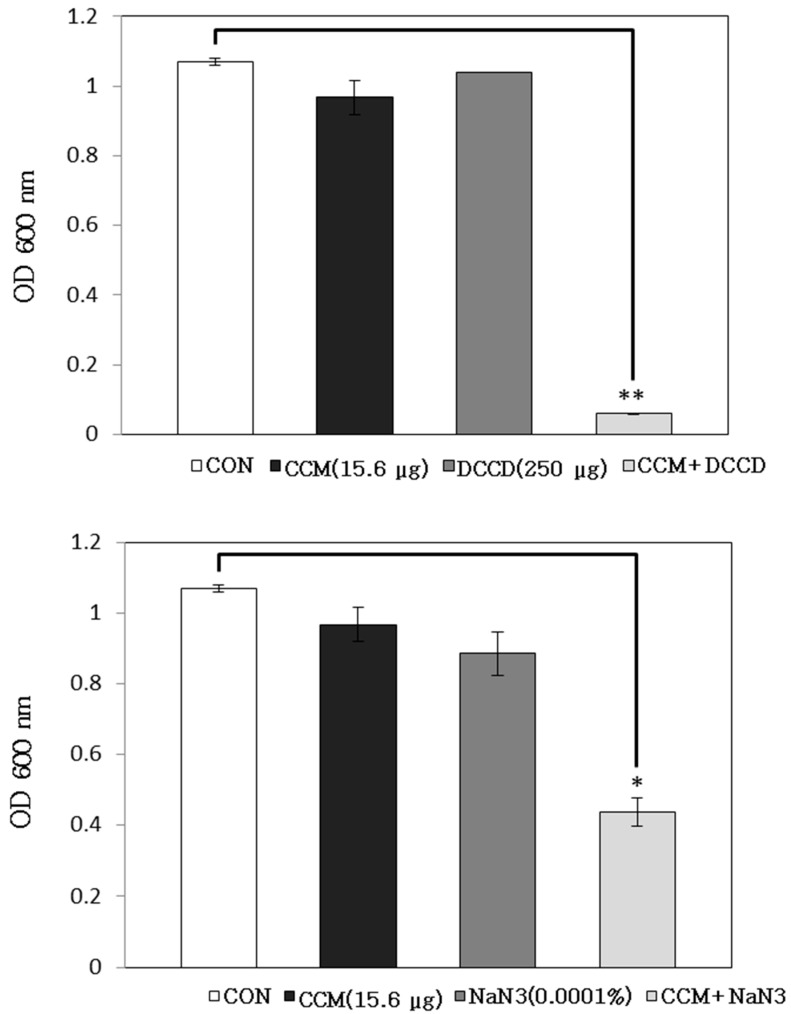
The effects of the ATPase-inhibitors, *N,N'*-dicyclohexylcarbodiimide (DCCD) and sodium azide (NaN_3_), on the susceptibility of *Staphylococcus aureus* (ATCC 33591) to curcumin (CCM) treatment. The viability of bacteria was determined via spectrophotometry (optical density at 600 nm, OD_600_) after incubation for 24 h with 15.6 μg/mL CCM with 250 μg/mL DCCD and 0.0001% NaN_3_, respectively. These data represent the average of three independent experiments. * *p* < 0.05 and ** *p* < 0.005, as compared to CCM alone, were determined. CON is the acronym for the control *S. aureus* strain, which was not treated.

ATP-binding cassette (ABC) transporters are located in the periplasm of Gram-negative bacteria and the cell surface of Gram-positive bacteria [16]. ABC transporters have ATP-dependent transporting activity, and the ATPase inhibitor, *N,N'*-dicyclohexylcarbodiimide (DCCD), inhibits the H+ translocation activity of the F_0_ domain of F_0_F_1_-ATPase that generates a proton motive force in the presence of agents, such as and sodium azide (NaN_3_) [17]. The combination of curcumin with ATPase inhibitors, DCCD and NaN_3_, showed a valid effect on the OD value. The bacterial viability in the presence of curcumin with 250 μg/mL DCCD and 0.0001% NaN_3_ reduced to 94% and 55%, respectively, with respect to the OD values of curcumin alone (Figure 3). We discovered that the antibacterial activity of curcumin was seriously affected by the cytoplasmic membrane permeability and ABC transporter, namely when curcumin is effectively used in combination with detergents or ATPase inhibitors to treat MRSA-infections.

### 2.2. Activity of Curcumin by Binding of Peptidoglycan

The cell walls of most Gram-positive bacteria consist mainly of numerous layers (up to 30) of glycan, with amino acid bridges connecting each layer to other layers above and below it. Lipopolysaccharide (LPS) is only attached to the outer membrane of the two layers in Gram-negative bacteria [18,19,20].

**Figure 4:**
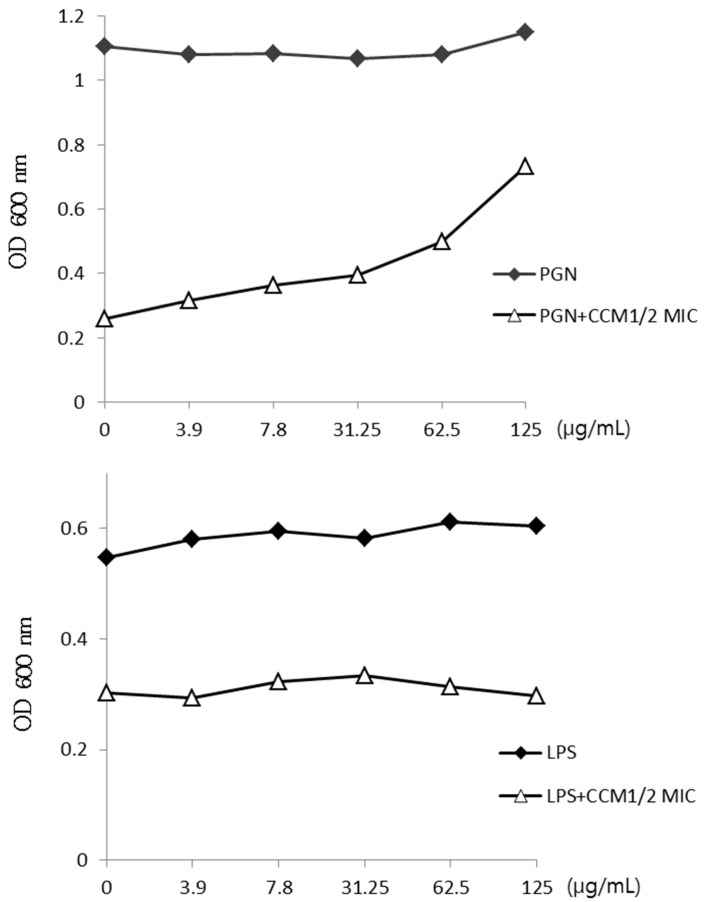
The binding of curcumin (CCM) with peptidoglycan (PGN) of the cell wall. PGN from *Staphylococcus aureus* (*S. aureus*) was added to Mueller–Hinton broth (MHB) containing CCM. Lipopolysaccharide (LPS) was used as a control.

Curcumin may bind to the cell wall and interrupt its integrity. The binding of curcumin with peptidoglycan (PGN) was confirmed upon the increasing concentration of PGN of *S. aureus* (Figure 4). At a concentration of 0–125 μg/mL, PGN gradually impeded the activity of curcumin. However, PGN did not show complete blocking with the antibacterial activity of curcumin. As a control, LPS did not show any variation at the same concentration.

### 2.3. Curcumin Reduces PBP2a Levels in MRSA

**Figure 5:**
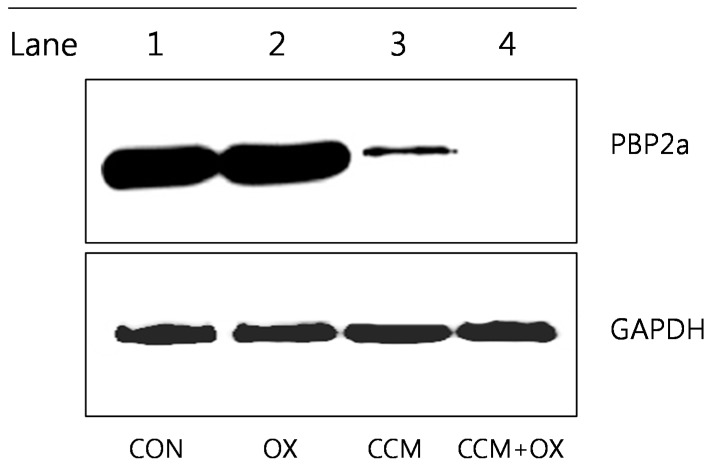
Western blot analysis of penicillin binding protein (PBP)2a production by ATCC 33591 after treatment with 32 µg/mL oxacillin (OX) and 250 µg/mL curcumin (CCM). The control (CON) was used without treatment drugs.

We expected that curcumin affects the level of PBP2a protein, encoded by the gene, *mecA*. As supposed, the western blot analysis indicated that curcumin reduced the secretion of PBP2a from MRSA (ATCC 33591), as shown in Figure 5. This level of PBP2a was induced further by 32 μg/mL oxacillin (OX); however, it was reduced by the addition of curcumin (250 μg/mL). The PBP2a protein was not present in the combination of curcumin and OX. This affected PBP2a level may indicate that curcumin interrupts the process of protein synthesis, by damaging RNA. Loading differences were normalized with monoclonal anti-GAPDH antibody.

**Figure 6:**
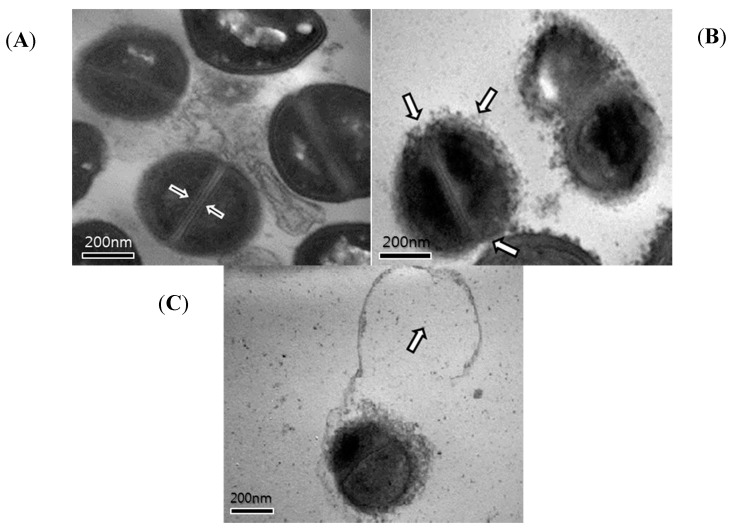
Transmission electron microscopy (TEM) images of methicillin-resistant *Staphylococcus aureus* (MRSA) (**A**–**C**) after 24 h of curcumin treatment. (A) Untreated control MRSA. These arrows indicate intact septa. (B) MRSA treated with 1/2 MIC of curcumin (125 µg/mL). These arrows indicate damage of cytoplasmic membrane. (C) MRSA treated with the MIC of curcumin (250 µg/mL). This arrow indicates a dividing cell following exposure to curcumin.

### 2.4. Transmission Electron Microscopy (TEM)

Some antimicrobial agents cause membrane damage and induce cell lysis; transmission electron microscopy (TEM) results have shown the impact of curcumin on the cell walls and cell membranes of MRSA (Figure 6). Another similar result has been reported, which confirmed the crumbly nature of the cell wall after treatment of *S. aureus* with antibacterial mineral leachates [21]. Other similar results have been reported, which demonstrated burst cell walls after treatment of MRSA with grape seed extract [22]. Cells are protected from lysis in low osmotic atmospheres owing to the high tensile strength cell wall, and such damage to the cell wall certainly decreases the tolerance of the cells to high ionic strengths and low osmotic pressures [23].

Changes in the ultrastructure of cells following exposure to curcumin was confirmed by TEM. The TEM images of the untreated MRSA strains confirmed intact septa. Following 8 h of exposure to 1/2 MIC of curcumin, damage of the cytoplasmic membrane was observed. The MIC of the curcumin-exposed MRSA cells was observed in cytoplasmic disruption and separation. These ultrastructural changes are indicative of cell lysis according to the antimicrobial activity of curcumin against MRSA.

## 3. Experimental Section

### 3.1. Isolation and Purification of Curcumin

Dried and pulverized rhizomes of *Curcuma longa* L. (600 g), purchased from the University Oriental herbal drugstore, were subjected twice to ethyl alcohol (2 L) extraction for 24 h at room temperature, to generate an extract (51.63 g). The extract was dissolved in 60% aqueous methanol (1 L) and partitioned with n-hexane (1 L × 3) followed by chloroform (1 L × 3). The 60% aqueous methanol layer was removed and evaporated in vacuum, and the resulting residue was dissolved in water and successively partitioned with normal butanol (1 L × 3). A portion (10 g) of the bioactive chloroform-soluble extract was subjected to silica-gel column chromatography and eluted with a chloroform/methanol mixture (80:1 to 16:1 in a step-wise gradient) to yield curcumin (2.21 g). The isolated curcumin was identified by the comparison of mass spectroscopy, ^1^H-NMR and ^13^C-NMR spectra, with those reported previously [24]. Curcumin (>95%) was deposited at the Standardized Material Bank for New Botanical Drugs (No. NNMBP000293-1) at Wonkwang University (Iksan, Republic of Korea).

### 3.2. Bacterial Strains and Growth Conditions

Among the six strains of *S. aureus* used in this study, four clinical MRSA isolates were obtained from four different patients at Wonkwang University Hospital. The remaining two strains, *S. aureus* ATCC 33591 (methicillin-resistant strain) and *S. aureus* ATCC 25923 (methicillin-susceptible strain, MSSA), were purchased form American Type Culture Collection (Manassas, VA, USA). All bacteria were stored in 30% glycerol and frozen at −70 °C before use. The bacterial strains were suspended in Mueller–Hinton broth (MHB) and incubated at 37 °C for 24 h.

### 3.3. Antimicrobial Resistance Testing

Detection of the *mecA* gene in MRSA strains was performed by PCR (polymerase chain reaction) amplification (Table 1). Prior to DNA extraction, bacteria stock cultures were subcultured twice onto MHA plates. For rapid extraction, one to five bacterial colonies were suspended in 300 μL of cell lysis buffer and heated at 100 °C for 20 min. After centrifugation at 12,000 rpm for 10 min, 2 μL of the supernatant were used for the DNA extraction. PCR reactions were performed using a MRSA Primer Mix Kit (Genotek, Daejeon, Republic of Korea). The PCR amplification consisted of 30 cycles (94 °C, 60 s; 55 °C, 60 s; 72 °C, 60 s). The final PCR products were separated on a 2% agarose gel.

**Table 1:** Determination of the *mecA* gene status of the *Staphylococcus aureus* strains used in this study.

*S. aureus*	Class	*mecA* Gene	β-Lactamase Activity	Antibiotic Resistance
ATCC 33591	MRSA	+	+	AM, OX
ATCC 25923	MSSA	−	−	−

+, positive; −, negative; AM, ampicillin; OX, oxacillin.

### 3.4. Reagents

Triton X-100 (TX-100), *N,N'*-dicyclohexylcarbodiimide (DCCD), purified lipopolysaccharide (LPS), paraformaldehyde, glutaraldehyde, sodium cacodylate buffer, osmium tetroxide, uranyl acetate, ethanol, propylene oxide and Spurr’s resin were purchased from Sigma-Aldrich Co. (St. Louis, MO, USA) Difco™ MHB was used as the nutrient media. *Tris*-(hydroxymethyl) aminomethane (Tris) was purchased from AMRESCO. Sodium azide (NaN_3_) and peptidoglycan (PGN) were purchased from Fluka. Bacterial protein extraction solution was purchased from Intron bio technology, Inc. (Seongnam, Korea).

### 3.5. Antibacterial Activity with Detergent or ATPase Inhibitors

To elucidate whether the antibacterial activity of curcumin was associated with the altered membrane permeability or the action of adenosine triphosphatase (ATPase), we examined the antibacterial activity of curcumin in the presence of detergents and ATPase-inhibiting agents. To determine the detergent-induced permeabilization, a particular concentration of curcumin was determined using detergents, TX-100 and Tris [25]. The non-ionic detergent TX-100 significantly increases antibiotic sensitivity [26]. DCCD and NaN_3_, metabolic inhibitors that can decrease ATP levels by disrupting electrochemical proton gradients in a bacterial environment, were used as an inhibitor of ATPase [27,28]. The antibacterial activity of curcumin was measured in the presence of 0.001% TX-100, 125 μg/mL Tris, 250 μg/mL DCCD and 0.0001% NaN_3_.

### 3.6. Effect of Exogenous Peptidoglycan on Curcumin Activity

To determine the activity of exogenous PGN in the presence of curcumin, and curcumin and PGN, combination assays were performed following the method described by Zhao *et al.* [29]. These assays indicated whether PGN blocks the activity of curcumin and curcumin interrupts the integrity of the cell wall. Curcumin was added to concentrated PGN, and the resulting solution was serially diluted. LPS was used as a control.

### 3.7. Western Blotting

The MRSA culture (ATCC 33591) was grown at OD_600_ of 0.4 in MHB for western blot analysis [30]. The cellular protein extracts were prepared from the bacterial harvest cells collected after 0.5 h of the treatment. Pellets were suspended in lysis buffer containing Tris/HCI (pH 7.5), and separated soluble protein was extracted by centrifugation at 13,000 rpm for 5 min. Western blot analysis was performed by using mouse anti-PBP2a antibody.

### 3.8. Transmission Electron Microscopy (TEM)

MRSA exponential phase cultures were prepared by diluting cultures in MHB overnight, and the cell growth was continued at 37 °C until the cultures reached the mid-logarithmic phase of growth. The MHB-grown exponential-phase MRSA was treated with 1/2 × MIC and 1 × MIC of curcumin for 8 h. Following the treatment, 2 mL of the culture was collected by centrifugation at 10,000 *g* for 10 min. After the removal of the supernatant, pellets were fixed with modified Ki Woo Kim fixative [31]. The specimens were examined using an energy-filtering transmission electron microscope (LIBRA 120; Carl Zeiss, Oberkochen, Germany) operated at an accelerating voltage of 120 kV. Transmitted electron signals were recorded using a 4 k × 4 k slow-scan charge-coupled device camera (Ultrascan 4000 SP; Gatan, Pleasanton, CA, USA) attached to an electron microscope.

## 4. Conclusions

In this study, we focused on the mechanism of the antimicrobial action of curcumin for the susceptibility of MRSA by viability assays and western blotting. The outcome shows that in developing natural antimicrobial agents against multidrug-resistant strains, this study on the mechanism of antimicrobial activity of curcumin may be potentially invaluable. Further, additional *in vivo* experiments are necessary for effective treatment.
